# Effect of the COVID-19 pandemic on potential health emergencies in paediatric patients: a retrospective cohort study

**DOI:** 10.3389/fpubh.2024.1402525

**Published:** 2024-09-09

**Authors:** Krzysztof Marek Mitura, Daniel Celiński, Jadwiga Snarska, Sławomir Dariusz Szajda

**Affiliations:** ^1^Independent Public Health Care Center RM-MEDITRANS Emergency Station and Sanitary Transport in Siedlce, Siedlce, Poland; ^2^Department of Emergency Medical Service, Medical University of Warsaw, Warsaw, Poland; ^3^Department of Surgery, Collegium Medicum, University of Warmia and Mazury in Olsztyn, Olsztyn, Poland; ^4^Department of Emergency Medical Service, Collegium Medicum, University of Warmia and Mazury in Olsztyn, Olsztyn, Poland

**Keywords:** health emergency, COVID-19, pandemic, paediatric patient, child, adolescent

## Abstract

The COVID-19 pandemic compromised the principles underlying the functioning of public health, which is understood as the prevention of diseases and care for the health of entire communities. During the pandemic period, the efforts of the health system focused on patients with suspected infection and those infected with the SARS-CoV-2 virus, which led to changes in the provision of health services and the characteristics of patients receiving medical services at the pre-hospital stage. The objective of this study was to investigate the effects of the COVID-19 pandemic on potential health emergencies in paediatric patients based on the International Statistical Classification of Diseases and Related Health Problems (ICD-10). The data used in the study were derived from interventions carried out by Emergency Medical Teams (EMT) in central and eastern Poland, involving patients who were under 18 years of age (*n* = 12,619). The data were collected from 1 January 2017 to 31 December 2022. The study used descriptive statistics, the Mann–Whitney U Test, and the Chi-square test. The study reveals that fewer paediatric patients (5.28%) were provided medical services by EMTs during the COVID-19 pandemic compared to the pre-pandemic period (5.86%). There was a decrease in the number of injuries in paediatric patients (from 42.0 to 32.7%; *p* < 0.001), and more patients were left at the location from which the call was made (18.9 vs. 23.9%; *p* < 0.001). Moreover, during the pandemic, as compared to the pre-pandemic period, there was an increase in the number of cases of pre-hospital assistance provided to paediatric patients with fever, irrespective of gender, area (village, city) or patient age. During the pandemic, paediatric patients consumed alcohol more frequently. The age of patients who were provided with assistance by EMTs decreased (median of 10.0 vs. 9.0; *p* < 0.001). The COVID-19 pandemic brought about changes in the prevalence of potential health emergencies in children. The incidence of injuries decreased, while the number of interventions due to fever and alcohol consumption increased. There was a reduction in the number of patients transported to the hospital. In addition, the age of patients who received medical assistance decreased. The study shows health problems that were faced by paediatric patients during the COVID-19 pandemic and, therefore, can be helpful in preparing the healthcare system for emergency situations.

## 1 Introduction

The emergence of the severe acute respiratory syndrome coronavirus 2 (SARS-CoV-2) in 2019 triggered a COVID-19 (coronavirus disease 2019) pandemic that became the greatest global public health hazard of the 21st century. The pandemic had a huge impact on numerous aspects of social life. The spread of the SARS-CoV-2 virus had an impact on the global economy as well as the availability and quality of healthcare services (1). Concerns about the spread of the virus resulted in changes in the organisation of the provision of health services, both in the fields of primary health care and inpatient health services. The implemented restrictions, aimed at ensuring the safety of medical staff and patients and concerning the organisation of healthcare facilities, significantly contributed to limiting the spread of the virus (2). Nonetheless, the COVID-19 pandemic disrupted the healthcare system, causing a deterioration in the health status of the population, especially individuals suffering from chronic diseases, as a result of public concerns about visiting a doctor and limited accessibility to healthcare services in the healthcare system focused on the COVID-19 pandemic (3, 4). At the same time, during the COVID-19 pandemic, as compared to the pre-pandemic period, there was a deterioration in the quality of healthcare services provided to patients (4).

During the pandemic, all the organisational structures of the healthcare sector faced a number of unprecedented challenges. The challenges, although sometimes converging, varied depending on the type of health care services provided by particular units. The restrictions and organisational changes applied in the healthcare sector during the COVID-19 pandemic resulted in patients searching for medical care, which had an impact on the functioning of pre-hospital emergency response teams (5). Thus, emergency medical teams, although obliged to provide medical assistance in the event of a medical emergency, also had to provide services resulting from the emergency situation caused by the pandemic. At the same time, it should be noted that a medical emergency should be understood as a sudden or shortly anticipated emergence of symptoms indicating deterioration of health, the immediate consequences of which may be a serious impairment of bodily functions, bodily harm or loss of life (6).

Since health emergencies are observed in patients of all ages, they also occur in individuals under 18 years of age. Regardless of the pandemic’s emergence, the most common cause of morbidity in paediatric patients (under 18 years of age) is respiratory diseases (7, 8). Injuries, on the other hand, are the main cause of death and disability among children, with the main mechanism of injury being motor vehicle collisions for children aged 10–18 years and falls from height for children under 9 years of age (9). At the same time, the most common cause of injury-related hospitalisation of children at Accident and Emergency Units is head injuries (10, 11).

The occurrence of the COVID-19 pandemic and the methods applied to prevent the spread of the virus, namely restrictions on social life (lockdown, remote learning), could have affected the health of children and adolescents. However, the incidence of SARS-CoV-2 virus infection itself, as well as the mortality rate as compared to adults, is low in these children, with children under 1 year of age being at risk of a very severe course of infection, especially in the case of co-morbidities (12).

The main objective of this study is to investigate the effect of the COVID-19 pandemic on the health of children and adolescents up to the age of 18 years based on pre-hospital medical interventions carried out by Emergency Medical Teams (EMT) in situations of the occurrence of a potential health emergency in paediatric patients, as compared to the pre-pandemic period. By health emergencies should be understood all EMT interventions, potential situations in which serious disorders of the human body, bodily harm or even death are expected to occur suddenly or within a short period of time (6).

## 2 Materials and methods

### 2.1 Study design

For The study was conducted based on the data derived from interventions carried out by EMTs in central and eastern Poland. The study area covers an area of 7,350 km2 and has a population of approximately 532,000, with people under 18 years of age accounting for 20.65% (*n* = 109,885) of the total population of the area (13). The retrospective cohort analysis was conducted on paediatric patients who had a potential health emergency between 1 January 2017 and 31 December 2022. In order to compare the period before the pandemic to the period of the COVID-19 pandemic, the time range under study was divided into sub-periods, one from 01 January 2017 to 31 December 2019 (the pre-pandemic period) and the second from 01 January 2020 to 31 December 2022 (the pandemic period). The start of the pandemic period was set at 01 January 2020 due to the difficulty in clearly identifying the time limits of the pandemic, the dynamic of the virus spread, and the initial difficulties in diagnosing infected patients. It should be noted that the COVID-19 disease was first diagnosed in November 2019 in the city of Wuhan, China, and as early as 11 March 2020, The World Health Organization declared a COVID-19 pandemic. In Poland, the first official case of COVID-19 infection was noted on 4 March 2020. On 14 March 2020, an epidemic emergency state was declared, while on 20 March 2020, a state of epidemic was announced.

### 2.2 Data

The study used data from the nationwide State Emergency Medical Service Command Support System, which is the central register of EMT interventions in Poland. The data used in the study only refer to patients under 18 years of age.

For each patient, the collected data included the age, gender, the area of EMT intervention (city, village), time of intervention, the diagnosis made according to the 3-character ICD-10 codes, and the result of the intervention. The diagnoses under analysis were limited to those that collectively accounted for at least 50% of the most frequent diagnoses involving a particular group during the period of 2017–2022. Moreover, the analyses that were conducted only concerned the pre-hospital management of patients.

The study was approved by the Research Ethics Committee of the University of Warmia and Mazury in Olsztyn (decision No. 20/2024).

### 2.3 Statistical analysis

The acquired data were stored in a database of the program Microsoft Excel MS Office 2021 for Windows 11. For the performance of statistical analyses and the visualisation of their results, the study used R ver. 4.3.1 (14) with the RStudio environment ver. 2023.09.1.494 (15), and the tidyverse package ver. 2.0.0 (16).

The study applied the Mann–Whitney U Test and the Chi-square test. In order to compare the age of patients (in years) between the groups under study, the Mann–Whitney non-parametric U test (Wilcoxon test - independent samples) and descriptive statistics, i.e., mean (M), median (Me), standard deviation (SD) and interquartile range (IQR), were applied. To investigate the relationships between the qualitative variables, the Chi-squared test (χ2) was applied, and in their description, the numerosity (n) and percentage (%) were used. The results were considered statistically significant at *p* < 0.05.

## 3 Results

From 1 January 2017 to 31 December 2022, EMTs carried out 226,039 interventions in central and eastern Poland, of which 5.58% (*n* = 12,619) were interventions for patients up to 18 years of age (paediatric patients). During the pre-pandemic period (the years 2017–2019), interventions to paediatric patients accounted for 5.86% (*n* = 6,827) of the 116,425 interventions recorded, and during the pandemic (the years 2020–2022), for 5.28% (*n* = 5,792) of the 109,614 interventions recorded. In addition, during the pandemic, 53 interventions were recorded which made a diagnosis related to being infected by the COVID-19 *U07.1-COVID-19 identified* (*n* = 48) or *U07.2-COVID-19 unidentified* (*n* = 5), which accounts for 0.048% of all interventions to paediatric patients in the years 2020–2022.

**Figure 1:**
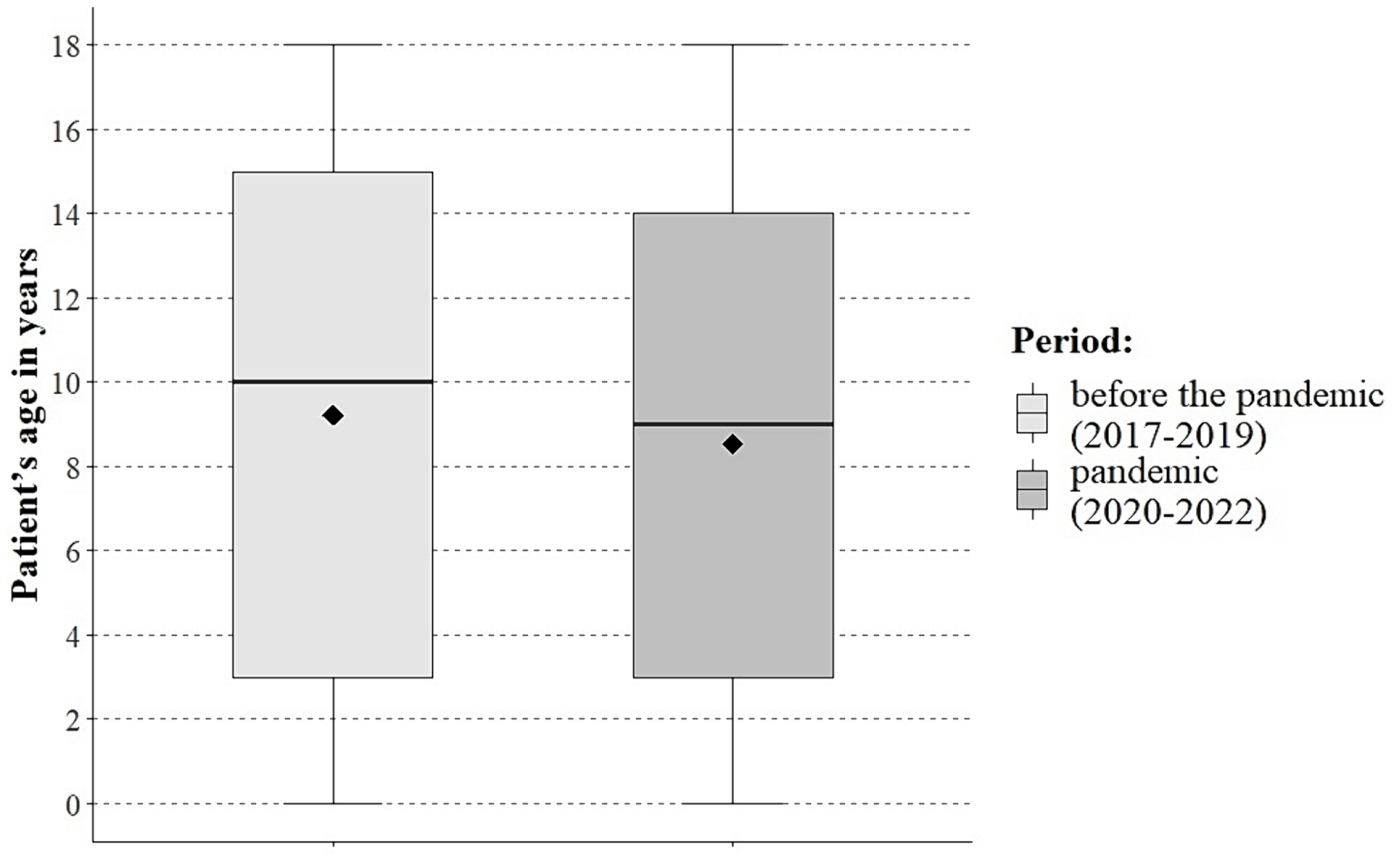
Patient characteristics.

No differences were noted between the periods under study in the number of EMT interventions in urban and rural areas or depending on the patient’s gender. A significant (*p* < 0.001) decrease in the paediatric patients’ age during the pandemic was noted, as compared to the pre-pandemic period (mean of 8.5 vs. 9.2, median of 9.0 vs. 10.0; Figure 1). During the pandemic, the percentage of interventions to patients between 1 and 4 years of age increased significantly (31.2 vs. 25.4%) and decreased in the age group 12 to 18 years (40.0% vs. 44.1%) to the pre-pandemic period. In the years 2020–2022, as compared to the years 2017–2019, there was a significant reduction in the percentage of EMT interventions due to injury (32.7% vs. 42.0%) and an increase in EMT interventions that ended with paediatric patients being left at the location from which the call had been made (23.9% vs. 18.9%) and decreased the percentage of paediatric patients transported to the hospital (75.9% vs. 80.8%). The full data are provided in Table 1.

**Table 1:** Patient characteristics.

Variable	Parameter	Period		*p*-value
		Before the pandemic (2017–2019)	Pandemic (2020–2022)	
		n (%)	n (%)	
Area of intervention	City	2,746 (40.2)	2,298 (39.7)	*p* = 0.531
	Village	4,081 (59.8)	3,494 (60.3)	
Gender	Female	3,643 (53.4)	3,133 (54.1)	*p* = 0.412
	Male	3,184 (46.6)	2,659 (45.9)	
Age	n	6,827	5,792	*p* < 0.001
	Mean (SD)	9.2 (5.8)	8.5 (5.9)	
	Median (IQR)	10.0 (3.0–15.0)	9.0 (3.0–14.0) ↓	
	Range	0–18	0–18	
Age range	up to 1 year of age	353 (5.2)	279 (4.8)	*p* = 0.364
	1 to 4	1,733 (25.4)	1,806 (31.2) ↑	*p* < 0.001
	5 to 11	1,727 (25.3)	1,390 (24.0)	*p* = 0.092
	from 12 to 18	3,014 (44.1)	2,317 (40.0) ↓	*p* < 0.001
Cause of intervention	Injury	2,870 (42.0)	1,893 (32.7) ↓	*p* < 0.001
	Incident case	3,957 (58.0)	3,899 (67.3) ↑	
Result of intervention	Transport	5,519 (80.8)	4,397 (75.9) ↓	*p* < 0.001
	Left at the place of call	1,292 (18.9)	1,384 (23.9) ↑	*p* < 0.001
	Death	16 (0.2)	11 (0.2)	*p* = 0.590

Between the periods under analysis, significant differences were noted in the number of EMT interventions for paediatric patients depending on the months: January (*p* = 0.041), March (*p* < 0.001), May (*p* = 0.034), June (*p* = 0.005), July (*p* = 0.039), November (*p* = 0.039), days: Tuesday (*p* = 0.046), Sunday (*p* = 0.046) and the hourly range of interventions:00:00–03:59 AM (*p* < 0.001), 04:00 AM–07.59 AM (*p* = 0.026), 08:00 AM–11:59 AM (*p* < 0.001), 12:00 PM–03:59 PM (*p* < 0.001), 08:00 PM–11:59 PM (*p* < 0.001). The greatest difference in the percentage of interventions between the pre-pandemic period and the pandemic period was noted for the months of March (8.4% vs. 10.6%) and June (9.1% vs. 10.6%), day Tuesday (14.9% vs. 13.7%) and Sunday (11.9% vs. 13.1%), while for the hourly ranges, for the periods of 08:00 PM–11:59 PM (15.5% vs. 19.4%) and 12:00 AM–03:59 PM (27.0% vs. 23.3%). The data covering the EMT interventions for paediatric patients depending on the variables, including the time of intervention, are provided in Table 2.

**Table 2:** EMT interventions depending on the month, day and hourly range.

Variable	Parameter	Period		*p*-value
		Before the pandemic (2017–2019)	Pandemic (2020–2022)	
		n (%)	n (%)	
Month	January	583 (8.5)	437 (7.5) ↓	*p* = 0.041
	February	508 (7.4)	434 (7.5)	*p* = 0.911
	March	575 (8.4)	614 (10.6) ↑	*p* < 0.001
	April	522 (7.6)	398 (6.9)	*p* = 0.095
	May	659 (9.7)	496 (8.6) ↓	*p* = 0.034
	June	623 (9.1)	614 (10.6) ↑	*p* = 0.005
	July	482 (7.1)	465 (8.0) ↑	*p* = 0.039
	August	469 (6.9)	417 (7.2)	*p* = 0.469
	September	592 (8.7)	507 (8.8)	*p* = 0.870
	October	681 (10.0)	530 (9.2)	*p* = 0.117
	November	550 (8.1)	410 (7.1) ↓	*p* = 0.039
	December	583 (8.5)	470 (8.1)	*p* = 0.389
Day	Monday	1,079 (15.8)	844 (14.6)	*p* = 0.054
	Tuesday	1,020 (14.9)	793 (13.7) ↓	*p* = 0.046
	Wednesday	996 (14.6)	874 (15.1)	*p* = 0.430
	Thursday	1,053 (15.4)	902 (15.6)	*p* = 0.817
	Friday	1,061 (15.5)	890 (15.4)	*p* = 0.786
	Saturday	805 (11.8)	731 (12.6)	*p* = 0.155
	Sunday	813 (11.9)	758 (13.1) ↑	*p* = 0.046
Hourly range	00:00–03:59 AM	428 (6.3)	486 (8.4) ↑	*p* < 0.001
	04:00 AM - 07.59 AM	343 (5.0)	343 (5.9) ↑	*p* = 0.026
	08:00 AM - 11:59 AM	1,632 (23.9)	1,193 (20.6) ↓	*p* < 0.001
	12:00 PM - 03:59 PM	1,841 (27.0)	1,351 (23.3) ↓	*p* < 0.001
	04:00 PM - 07:59 PM	1,522 (22.3)	1,293 (22.3)	*p* = 0.967
	08:00 PM - 11:59 PM	1,061 (15.5)	1,126 (19.4) ↑	*p* < 0.001

Table 3 shows a comparison of EMT interventions covering 50% of the most frequently given diagnoses during the period 2017–2022, which indicates that during the COVID-19 pandemic, as compared to the pre-pandemic period, there was a significant increase in the percentage of patients with the following diagnoses: *R50, Fever of other and unknown origin* (10.0% vs. 6.9%), *Z04, Examination and observation for other reasons* (4.0% vs. 2.1%), *R11, Nausea and vomiting* (3.0% vs. 2.4%), and a significant decrease in the percentage of patients with the following diagnoses: *S00, Superficial injury of the head* (6.0% vs. 8.2%), *R55, Syncope and collapse* (7.6% vs. 8.7%), *S01, Open wound of the head* (3.5% vs. 4.4%), and *S09, Other and unspecified injuries of the head* (1.3% v 2.2%).

**Table 3:** EMT interventions in the pre-pandemic period (2017–2019) and during the pandemic (2020–2022).

ICD-10*	Years 2017–2022	Period		*p*-value
		Before the pandemic (2017–2019)	Pandemic (2020–2022)	
	n (%)	n (%)	n (%)	
R50	1,055 (8.4)	473 (6.9)	582 (10.0) ↑	*p* < 0.001
R55	1,035 (8.2)	597 (8.7)	438 (7.6) ↓	*p* = 0.016
S00	908 (7.2)	562 (8.2)	346 (6.0) ↓	*p* < 0.001
R10	630 (5)	327 (4.8)	303 (5.2)	*p* = 0.256
S01	498 (3.9)	297 (4.4)	201 (3.5) ↓	*p* = 0.011
R56	481 (3.8)	254 (3.7)	227 (3.9)	*p* = 0.561
Z03	369 (2.9)	140 (2.1)	229 (4.0) ↑	*p* < 0.001
R06	344 (2.7)	182 (2.7)	162 (2.8)	*p* = 0.652
R11	335 (2.7)	162 (2.4)	173 (3.0) ↑	*p* = 0.033
S09	225 (1.8)	149 (2.2)	76 (1.3) ↓	*p* < 0.001
Z00	221 (1.8)	126 (1.8)	95 (1.6)	*p* = 0.381
G40	214 (1.7)	107 (1.6)	107 (1.8)	*p* = 0.225
Other	6,304 (50.0)	3,451 (50.5)	2,853 (49.3)	*p* = 0.148
Total	12,619 (100)	6,827 (100)	5,792 (100)	

*G40, Epilepsy; R06, Abnormalities of breathing; R10, Abdominal and pelvic pain; R11, Nausea and vomiting; R50, Fever of other and unknown origin; R55, Syncope and collapse; R56, Convulsions, not elsewhere classified; S00, Superficial injury of the head; S01, Open wound of the head; S09, Other and unspecified injuries of the head; Z00, General examination and investigation of persons without complaint and reported diagnosis; Z03, Medical observation and evaluation for suspected diseases and conditions ruled out.

A comparison of EMT interventions in the urban area (Table 4), covering 50% of the diagnoses most frequently given during the period 2017–2022, indicates a significant increase in the percentage of patients with the following diagnoses: *R50, Fever of other and unknown origin* (9.9% vs. 7.1%), *Z03, Medical observation and evaluation for suspected diseases and conditions, ruled out* (3.2% vs. 1.4%), *R11, Nausea and vomiting* (3.0% vs. 2.0%), and a significant decrease in the percentage of patients with the following diagnoses: *S00, Superficial injury of the head* (6.4% vs. 8.6%), *S01, Open wound of the head* (3.6% vs. 4.8%), and *Z04, Examination and observation for other reasons* (1.3% vs. 1.2%) during the COVID-19 period, as compared to the pre-pandemic period.

**Table 4:** EMT interventions in urban areas in the pre-pandemic period (2017–2019) and during the pandemic (2020–2022).

ICD-10*	Years 2017–2022	Urban area		*p*-value
		Before the pandemic (2017–2019)	Pandemic (2020–2022)	
	n (%)	n (%)	n (%)	
R55	467 (9.3)	265 (9.7)	202 (8.8)	*p* = 0.294
R50	422 (8.4)	195 (7.1)	227 (9.9) ↑	*p* < 0.001
S00	384 (7.6)	237 (8.6)	147 (6.4) ↓	*p* = 0.003
R10	243 (4.8)	140 (5.1)	103 (4.5)	*p* = 0.309
S01	215 (4.3)	133 (4.8)	82 (3.6) ↓	*p* = 0.026
R56	162 (3.2)	89 (3.2)	73 (3.2)	*p* = 0.897
R06	128 (2.5)	65 (2.4)	63 (2.7)	*p* = 0.400
R11	125 (2.5)	56 (2.0)	69 (3.0) ↑	*p* = 0.028
Z03	112 (2.2)	38 (1.4)	74 (3.2) ↑	*p* < 0.001
Z00	99 (2.0)	55 (2.0)	44 (1.9)	*p* = 0.822
Z04	91 (1.8)	60 (2.2)	31 (1.3) ↓	*p* = 0.026
G40	89 (1.8)	44 (1.6)	45 (2.0)	*p* = 0.339
Other	2,507 (49.7)	1,369 (49.9)	1,138 (49.5)	*p* = 0.814
Total	5,044 (100)	2,746 (100)	2,298 (100)	

*G40, Epilepsy; R06, Abnormalities of breathing; R10, Abdominal and pelvic pain; R11, Nausea and vomiting; R50, Fever of other and unknown origin; R55, Syncope and collapse; R56, Convulsions, not elsewhere classified; S00, Superficial injury of the head; S01, Open wound of the head; Z00, General examination and investigation of persons without complaint and reported diagnosis; Z03, Medical observation and evaluation for suspected diseases and conditions ruled out; Z04, Examination and observation for other reasons.

In rural areas, between 2017 and 2022, EMT interventions (Table 5) accounted in total for at least 50% of the most frequently given diagnoses. However, during the COVID-19 pandemic (Table 5), as compared to the pre-pandemic period, there was a significant increase in these interventions leading to the following diagnoses: *R50, Fever of other and unknown origin* (10.2% vs. 6.8%), *Z03, Medical observation and evaluation for suspected diseases and conditions, ruled out* (4.4% vs. 2.5%), *R10, Abdominal and pelvic pain* (5.7% vs. 4.6%), and a significant decrease in interventions resulting in the following diagnoses: *S00, Superficial injury of the head* (8.0% vs. 5.7%), *R55, Syncope and collapse* (6.8% vs. 8.1%) and *S09, Other and unspecified injuries of the head* (1.5% vs. 2.4%).

**Table 5:** EMT interventions in rural areas in the pre-pandemic period (2017–2019) and during the pandemic (2020–2022).

ICD-10*	Years 2017–2022	Rural area		*p*-value
		Before the pandemic (2017–2019)	Pandemic (2020–2022)	
	n (%)	n (%)	n (%)	
R50	633 (8.4)	278 (6.8)	355 (10.2) ↑	*p* < 0.001
R55	568 (7.5)	332 (8.1)	236 (6.8) ↓	*p* = 0.023
S00	524 (6.9)	325 (8.0)	199 (5.7) ↓	*p* < 0.001
R10	387 (5.1)	187 (4.6)	200 (5.7) ↑	*p* = 0.024
R56	319 (4.2)	165 (4.0)	154 (4.4)	*p* = 0.431
S01	283 (3.7)	164 (4.0)	119 (3.4)	*p* = 0.161
Z03	257 (3.4)	102 (2.5)	155 (4.4) ↑	*p* < 0.001
R06	216 (2.9)	117 (2.9)	99 (2.8)	*p* = 0.930
R11	210 (2.8)	106 (2.6)	104 (3.0)	*p* = 0.316
S09	152 (2.0)	99 (2.4)	53 (1.5) ↓	*p* = 0.005
G40	125 (1.7)	63 (1.5)	62 (1.8)	*p* = 0.432
Z00	122 (1.6)	71 (1.7)	51 (1.5)	*p* = 0.334
Other	3,779 (49.9)	2,072 (50.8)	1,707 (48.9)	*p* = 0.096
Total	7,575 (100)	4,081 (100)	3,494 (100)	

*G40, Epilepsy; R06, Abnormalities of breathing; R10, Abdominal and pelvic pain; R11, Nausea and vomiting; R50, Fever of other and unknown origin; R55, Syncope and collapse; R56, Convulsions, not elsewhere classified; S00, Superficial injury of the head; S01, Open wound of the head; S09, Other and unspecified injuries of the head; Z00, General examination and investigation of persons without complaint and reported diagnosis; Z03, Medical observation and evaluation for suspected diseases and conditions ruled out.

As shown in Table 6, a comparison of EMT interventions, covering 50% of the most frequently given diagnoses in female paediatric patients during the period of 2017–2022, indicates that during the COVID-19 pandemic period, as compared to the pre-pandemic period, there was a significant increase in the percentage of patients with the following diagnoses: *R50, Fever of other and unknown origin* (9.5% vs. 6.7%) and *Z03, Medical observation and evaluation for suspected diseases and conditions, ruled out* (4.2% vs. 2.7%), and a significant decrease in the percentage of patients with the following diagnoses: *R55, Syncope and collapse* (9.9% vs. 12.0%), *S00, Superficial injury of the head* (5.2% vs. 7.2%) and *S09, Other and unspecified injuries of the head* (1.2% vs. 2.1%).

**Table 6:** EMT interventions for girls in the pre-pandemic period (2017–2019) and during the pandemic (2020–2022).

ICD-10*	Years 2017–2022	Gender: female		*p*-value
		Before the pandemic (2017–2019)	Pandemic (2020–2022)	
	n (%)	n (%)	n (%)	
R55	645 (11.0)	381 (12.0)	264 (9.9) ↓	*p* = 0.013
R50	464 (7.9)	212 (6.7)	252 (9.5) ↑	*p* < 0.001
S00	367 (6.3)	229 (7.2)	138 (5.2) ↓	*p* = 0.002
R10	334 (5.7)	181 (5.7)	153 (5.8)	*p* = 0.909
R56	226 (3.9)	110 (3.5)	116 (4.4)	*p* = 0.073
Z03	198 (3.4)	87 (2.7)	111 (4.2) ↑	*p* = 0.002
S01	166 (2.8)	99 (3.1)	67 (2.5)	*p* = 0.177
R11	156 (2.7)	74 (2.3)	82 (3.1)	*p* = 0.073
R06	145 (2.5)	76 (2.4)	69 (2.6)	*p* = 0.611
Z00	112 (1.9)	62 (1.9)	50 (1.9)	*p* = 0.853
S09	98 (1.7)	67 (2.1)	31 (1.2) ↓	*p* = 0.005
S83	92 (1.6)	59 (1.9)	33 (1.2)	*p* = 0.061
Other	2,840 (48.6)	1,547 (48.6)	1,293 (48.6)	*p* = 0.975
Total	5,843 (100)	3,184 (100)	2,659 (100)	

*R06, Abnormalities of breathing; R10, Abdominal and pelvic pain; R11, Nausea and vomiting; R50, Fever of other and unknown origin; R55, Syncope and collapse; R56, Convulsions, not elsewhere classified; S00, Superficial injury of the head; S01, Open wound of the head; S09, Other and unspecified injuries of the head; S83, Dislocation, sprain and strain of joints and ligaments of the knee; Z00, General examination and investigation of persons without complaint and reported diagnosis; Z03, Medical observation and evaluation for suspected diseases and conditions ruled out.

On the other hand, a comparison of EMT interventions to boys, which accounted for at least 50% of the most common diagnoses for boys during the period 2017–2022 (Table 7), indicates that, during the COVID-19 pandemic period, compared to the pre-pandemic period, there was a significant increase in the percentage of patients with the following diagnoses: *R50 - Fever of other and unknown origin* (10.5% vs. 7.2%) and *Z03, Medical observation and evaluation for suspected diseases and conditions, ruled out* (3.8% vs. 1.5%), and a significant reduction in the percentage of the following diagnoses: *S00, Superficial injury of the head* (6.6% vs. 9.1%), *S01, Open wound of the head* (4.3% vs. 5.4%) and *S09, Other and unspecified injuries of the head* (1.4% vs. 2.3%).

**Table 7:** EMT interventions for boys in the pre-pandemic period (2017–2019) and during the pandemic (2020–2022).

ICD-10*	Years 2017–2022	Gender: male		*p*-value
		Before the pandemic (2017–2019)	Pandemic (2020–2022)	
	n (%)	n (%)	n (%)	
R50	591 (8.7)	261 (7.2)	330 (10.5) ↑	*p* < 0.001
S00	541 (8.0)	333 (9.1)	208 (6.6) ↓	*p* < 0.001
R55	390 (5.8)	216 (5.9)	174 (5.6)	*p* = 0.508
S01	332 (4.9)	198 (5.4)	134 (4.3) ↓	*p* = 0.028
R10	296 (4.4)	146 (4.0)	150 (4.8)	*p* = 0.117
R56	255 (3.8)	144 (4.0)	111 (3.5)	*p* = 0.377
R06	199 (2.9)	106 (2.9)	93 (3.0)	*p* = 0.887
R11	179 (2.6)	88 (2.4)	91 (2.9)	*p* = 0.211
Z03	171 (2.5)	53 (1.5)	118 (3.8) ↑	*p* < 0.001
G40	135 (2.0)	72 (2.0)	63 (2.0)	*p* = 0.919
J04	131 (1.9)	70 (1.9)	61 (1.9)	*p* = 0.939
S09	127 (1.9)	82 (2.3)	45 (1.4) ↓	*p* = 0.014
S52	113 (1.7)	63 (1.7)	50 (1.6)	*p* = 0.669
Other	3,316 (48.9)	1,811 (49.7)	1,505 (48.0)	*p* = 0.169
Total	6,776 (100)	3,643 (100)	3,133 (100)	

*G40, Epilepsy; J04, Acute laryngitis and tracheitis; R06, Abnormalities of breathing; R10, Abdominal and pelvic pain; R11, Nausea and vomiting; R50, Fever of other and unknown origin; R55, Syncope and collapse; R56, Convulsions, not elsewhere classified; S00, Superficial injury of the head; S01, Open wound of the head; S09, Other and unspecified injuries of the head; S52, Fracture of forearm; Z03, Medical observation and evaluation for suspected diseases and conditions ruled out.

As shown in Table 8, for all the EMT interventions, which covered at least 50% of the diagnoses most frequently given to patients aged up to 1 year during the period of 2017–2022, no significant differences concerning the period of the COVID-19 pandemic were noted, as compared to the pre-pandemic period.

**Table 8:** EMT interventions for patients aged up to 1 year in the pre-pandemic period (2017–2019) and during the pandemic (2020–2022).

ICD-10*	Years 2017–2022	Age: up to 1 year		*p*-value
		Before the pandemic (2017–2019)	Pandemic (2020–2022)	
	n (%)	n (%)	n (%)	
R06	65 (10.3)	38 (10.8)	27 (9.7)	*p* = 0.655
P24	64 (10.1)	35 (9.9)	29 (10.4)	*p* = 0.843
Z03	60 (9.5)	28 (7.9)	32 (11.5)	*p* = 0.132
R50	46 (7.3)	23 (6.5)	23 (8.2)	*p* = 0.406
Z00	40 (6.3)	18 (5.1)	22 (7.9)	*p* = 0.153
S00	35 (5.5)	24 (6.8)	11 (3.9)	*p* = 0.119
R11	28 (4.4)	15 (4.2)	13 (4.7)	*p* = 0.803
Other	294 (46.5)	172 (48.7)	122 (43.7)	*p* = 0.211
Total	632 (100)	353 (100)	279 (100)	

*P24, Neonatal aspiration syndromes; R06, Abnormalities of breathing; R11, Nausea and vomiting; R50, Fever of other and unknown origin; S00, Superficial injury of the head; Z00, General examination and investigation of persons without complaint and reported diagnosis; Z03, Medical observation and evaluation for suspected diseases and conditions ruled out.

A comparison of EMT interventions, which covered a minimum of 50% of the diagnoses most frequently given to paediatric patients aged from 1 year to 5 years during the period of 2017–2022 (Table 9), indicates that during the COVID-19 period, as compared to the pre-pandemic period, there was a significant increase in patients for the following diagnoses made: *R50, Fever of other and unknown origin* (21.8% vs. 18.3%) and *Z03, Medical observation and evaluation for suspected diseases and conditions, ruled out* (5.4% vs. 2.5%).

**Table 9:** EMT interventions for patients aged from 1 to 5 years in the pre-pandemic period (2017–2019) and during the pandemic (2020–2022).

ICD-10*	Years 2017–2022	Age: from 1 to 5 years		*p*-value
		Before the pandemic (2017–2019)	Pandemic (2020–2022)	
	n (%)	n (%)	n (%)	
R50	711 (20.1)	317 (18.3)	394 (21.8) ↑	*p* = 0.009
R56	278 (7.9)	142 (8.2)	136 (7.5)	*p* = 0.463
S00	249 (7.1)	129 (7.4)	120 (6.6)	*p* = 0.353
R11	186 (5.3)	87 (5.0)	99 (5.5)	*p* = 0.539
S01	147 (4.2)	76 (4.4)	71 (3.9)	*p* = 0.499
R06	142 (4.0)	77 (4.4)	65 (3.6)	*p* = 0.201
Z03	141 (4.0)	43 (2.5)	98 (5.4) ↑	*p* < 0.001
Other	1,685 (47.6)	862 (49.7)	823 (45.6) ↓	*p* = 0.013
Total	3,539 (100)	1,733 (100)	1,806 (100)	

*R06, Abnormalities of breathing; R11, Nausea and vomiting; R50, Fever of other and unknown origin; R56, Convulsions, not elsewhere classified; S00, Superficial injury of the head; S01, Open wound of the head; Z03, Medical observation and evaluation for suspected diseases and conditions ruled out.

On the other hand, a comparison of EMT interventions to patients aged from 5 to 12 years, which accounted for at least 50% of the most common diagnoses for boys during the period 2017–2022 (Table 10), indicates that during the COVID-19 pandemic period, compared to the pre-pandemic period, there was a significant increase in the percentage of patients with the following diagnosis: *R50, Fever of other and unknown origin* (9.3% vs. 5.4%) and a significant reduction in the percentage of patients with the following diagnosis: *S00, Superficial injury of the head* (7.5% vs. 10.8%).

**Table 10:** EMT interventions for patients aged from 5 to 12 years in the pre-pandemic period (2017–2019) and during the pandemic (2020–2022).

ICD-10*	Years 2017–2022	Age: 5 to 12 years		*p*-value
		Before the pandemic (2017–2019)	Pandemic (2020–2022)	
	n (%)	n (%)	n (%)	
S00	290 (9.3)	186 (10.8)	104 (7.5) ↓	*p* = 0.002
R10	237 (7.6)	119 (6.9)	118 (8.5)	*p* = 0.094
R50	223 (7.2)	94 (5.4)	129 (9.3) ↑	*p* < 0.001
S01	209 (6.7)	129 (7.5)	80 (5.8)	*p* = 0.057
R55	181 (5.8)	99 (5.7)	82 (5.9)	*p* = 0.843
R56	92 (3.0)	50 (2.9)	42 (3.0)	*p* = 0.836
S52	79 (2.5)	41 (2.4)	38 (2.7)	*p* = 0.525
G40	77 (2.5)	40 (2.3)	37 (2.7)	*p* = 0.537
S09	73 (2.3)	46 (2.7)	27 (1.9)	*p* = 0.186
J04	64 (2.1)	35 (2.0)	29 (2.1)	*p* = 0.907
Z03	64 (2.1)	34 (2.0)	30 (2.2)	*p* = 0.711
Other	1,528 (49.0)	854 (49.4)	674 (48.5)	*p* = 0.594
Total	3,117 (100)	1,727 (100)	1,390 (100)	

*G40, Epilepsy; J04, Acute laryngitis and tracheitis; R10, Abdominal and pelvic pain; R50, Fever of other and unknown origin; R55, Syncope and collapse; R56, Convulsions, not elsewhere classified; S00, Superficial injury of the head; S01, Open wound of the head; S09, Other and unspecified injuries of the head; S52, Fracture of forearm; Z03, Medical observation and evaluation for suspected diseases and conditions ruled out.

As shown in Table 11, a comparison of EMT interventions, covering 50% of the most frequently given diagnoses in patients aged from 12 to 18 years during the period of 2017–2022, indicates that during the COVID-19 pandemic period, as compared to the pre-pandemic period, there was a significant increase in the percentage of patients with the following diagnoses: *Z03, Medical observation and evaluation for suspected diseases and conditions, ruled out* (3.0% vs. 1.2%) and *Y91, Evidence of alcohol involvement determined by level of intoxication* (2.5% vs. 1.6%), and a significant decrease in the percentage of the following diagnoses: *R55, Syncope and collapse* (9.9% vs. 12.0%), *S00, Superficial injury of the head* (4.8% vs. 7.4%) and *S09, Other and unspecified injuries of the head* (0.9% vs. 2.2%).

**Table 11:** EMT interventions for patients aged from 12 to 18 years in the pre-pandemic period (2017–2019) and during the pandemic (2020–2022).

ICD-10*	Years 2017–2022	Age: 12 to 18 years		*p*-value
		Before the pandemic (2017–2019)	Pandemic (2020–2022)	
	n (%)	n (%)	n (%)	
R55	728 (13.7)	434 (14.4)	294 (12.7)	*p* = 0.071
S00	334 (6.3)	223 (7.4)	111 (4.8) ↓	*p* < 0.001
R10	289 (5.4)	152 (5)	137 (5.9)	*p* = 0.164
S83	174 (3.3)	95 (3.2)	79 (3.4)	*p* = 0.600
S01	135 (2.5)	86 (2.9)	49 (2.1)	*p* = 0.089
R07	107 (2.0)	60 (2.0)	47 (2.0)	*p* = 0.922
Y91	106 (2.0)	47 (1.6)	59 (2.5) ↑	*p* = 0.010
Z03	104 (2.0)	35 (1.2)	69 (3.0) ↑	*p* < 0.001
F99	101 (1.9)	49 (1.6)	52 (2.2)	*p* = 0.101
R51	101 (1.9)	61 (2.0)	40 (1.7)	*p* = 0.430
R56	97 (1.8)	51 (1.7)	46 (2.0)	*p* = 0.427
G40	91 (1.7)	46 (1.5)	45 (1.9)	*p* = 0.245
S09	87 (1.6)	65 (2.2)	22 (0.9) ↓	*p* < 0.001
S80	86 (1.6)	53 (1.8)	33 (1.4)	*p* = 0.337
R06	84 (1.6)	40 (1.3)	44 (1.9)	*p* = 0.097
R50	75 (1.4)	39 (1.3)	36 (1.6)	*p* = 0.425
Other	2,632 (49.4)	1,478 (49)	1,154 (49.8)	*p* = 0.578
Total	5,331 (100)	3,014 (100)	2,317 (100)	

*F99, Mental disorder, not otherwise specified; G40, Epilepsy; R06, Abnormalities of breathing; R07, Pain in throat and chest; R10, Abdominal and pelvic pain; R50, Fever of other and unknown origin; R51, Headache; R55, Syncope and collapse; R56, Convulsions, not elsewhere classified; S00, Superficial injury of the head; S01, Open wound of the head; S09, Other and unspecified injuries of the head; S80, Superficial injury of the lower leg; S83, Dislocation, sprain and strain of joints and ligaments of the knee; Y91, Evidence of alcohol involvement determined by the level of intoxication; Z03, Medical observation and evaluation for suspected diseases and conditions ruled out.

## 4 Discussion

This study addresses changes in the diagnoses made during the COVID-19 pandemic in the situation of a potential health emergency in paediatric patients in central and eastern Poland, which were recorded following an EMT intervention.

The research reveals that during the pandemic, there was a decrease in the number of EMT interventions for patients up to 18 years of age, from 5.28 to 5.86% (Table 1), as compared to the period preceding the COVID-19 pandemic. A similar situation was observed in Germany, particularly during periods when strict precautions were in place, which may be due, e.g., to lower morbidity resulting from changes in public behaviour during the pandemic, but also to the avoidance of medical services in the case of both less urgent and severe conditions (5, 17). A decrease in the number of EMT interventions during the COVID-19 pandemic was also noted in the United States (18). At the same time, a study of the emergency ambulance service in the state of Victoria, Australia, found that despite the smaller number of interventions, the ambulance response time, the duration of interventions, and the time taken to transfer patients to the hospital increased (19). During the pandemic, fewer EMT interventions were made for paediatric patients, while the children who did receive interventions were more seriously ill than during other times, according to Oulasvirta et al. (20). Besides the decrease in the number of EMT interventions to paediatric patients in Spain and Italy, a significant decrease was also noted in the number of paediatric visits to hospital accident and emergency departments and a significant decrease in the number of emergency admissions to hospitals (21, 22). On the other hand, a Dutch study indicated a significant decrease in the use of accident and emergency departments and hospitalisation of children, particularly in terms of visits to accident and emergency departments and hospital admissions due to infections that were not caused by the SARS-CoV-2 virus (23). A Polish study showed that patients of the emergency ambulance service and hospital accident and emergency departments positively assessed the operations of medical services during the COVID-19 pandemic (24).

This study suggests that paediatric patients who potentially experienced a health emergency during the pandemic were younger compared to the pre-pandemic period (mean of 8.5 vs. 9.2, median of 9.0 vs. 10.0; Table 1; Figure 1). Moreover, during the pandemic, there was an increase in the number of EMT interventions for patients between 1 and 4 years of age (31.2% vs. 25.4%), with a decrease noted for in the age group 12 to 18 years (40.0% vs. 44.1%) compared to the pre-pandemic period (Table 1). Similarly, a Finnish study indicates a decrease in paediatric patient age during the COVID-19 pandemic, as compared to the pre-pandemic period (20).

The current study also indicated changes in the use of medical care provided by EMTs for paediatric patients, depending on the month, the days of the week and the hourly range of the intervention (Table 2). A significant higher number of disease cases were noted during the pandemic in the months of March (10.6% vs. 8.4%), June (10.6% vs. 9.1%), July (8.0% vs. 7.1%) and reducing in January (7.5% vs. 8.5%), May (8.6% vs. 9.7%), November (7.1% vs. 8.1%). During the pandemic, there was a significant increase in the number of EMT interventions on Sunday (13.1% vs. 11.9%) and decreased on Tuesday (13.7% vs. 14.9%). However, for the hourly ranges, there was an increase in EMT interventions at night and in the morning (08:00 PM - 07.59 AM) and a decrease during the morning and midday hours (07:00 AM - 03:59 PM) during the pandemic. This may be indicative of the difficulty of accessing local outpatient care services at night and in the morning, the willingness to use medical assistance and the availability of outpatient clinics during their working hours, i.e., between the morning and midday hours.

In addition, it follows from the current study that in the years 2020–2022, as compared to the years 2017–2019, there was a decrease in the number of medical emergencies due to injury (32.7% vs. 42.0%; Table 1). This is most evident in the most common injuries noted in paediatric patients, i.e., different types of head injuries (Tables 3–7, 9–11). The reduction in the number of injuries among paediatric patients might have been due to the introduced pandemic-related restrictions on movement as well as lockdowns, e.g., of public institutions, including the closure of schools and the introduction of remote learning. A Canadian study by Keays et al. confirmed the current findings and indicated a reduction in the number of injuries, which was most evident for children aged from 6 to 11 years old (25). As for admissions of paediatric patients to hospital accident and emergency departments and trauma units in the United States, it was observed that the total number of admissions was significantly lower during the COVID-19 pandemic, as compared to the pre-pandemic period, which was due to the decrease in the number of traffic accidents, while the number of admissions due to burns and penetrating injuries increased (26). At the same time, Chaudhari et al. point out, based on Los Angeles data, that the severity of injuries and the Glasgow Coma Scale scores in children differed between the pre-pandemic and the pandemic period; however, mortality due to injuries decreased during the pandemic (27). Another study by Flynn-O’Brien et al. reported that during the first 6 months of the pandemic, the number of injuries in children, both unintentional and deliberate, increased by 20% (28).

It follows from the current study that during the pandemic, there was a significant increase in the number of medical care provided to patients aged from 1 to 12 years due to fever (*R50, Fever of other and unknown origin*; Tables 3–7, 9–10), irrespective of the gender and place of residence (village, city). This suggests that the increase in the number of medical services provided to patients with fever may be due to the knowledge of paediatric patient carers on the main symptoms associated with the SARS-CoV-2 virus infection. These typical symptoms, particularly at the beginning of the COVID-19 infection, include fever, cough, muscle aches and fatigue (29, 30). It should be noted that a sudden increase in EMT interventions due to a suspected infection may be an early warning sign of an increase in the number of positive COVID-19 test results a week later (31). At the same time, fever is the most common cause of unjustified EMT interventions for paediatric patients and their visits to hospital accident and emergency departments (32).

The current study showed that the COVID-19 pandemic, in addition to a number of mental and emotional problems observed in children and adolescents in relation to substance abuse, suicides, impaired social interaction and learning problems (33, 34), resulted in a significant increase in the number of medical interventions to children of the age group of 12 to 18 years, mainly caused by alcohol consumption, as compared to the pre-pandemic period (2.5% vs. 1.6%; Table 11). This may be due to the fact that isolation and quarantine, as well as preventive measures to limit the spread of the pandemic, such as social distancing and closure of schools, among others, adversely affected the mental health and well-being of children and adolescents (33, 35).

It was concluded that during the pandemic, as compared to the pre-pandemic period, there was a significant increase in the number of EMT interventions that ended with paediatric patients being left at the location from which the call had been made (23.9% vs. 18.9%) and decreased the percentage of paediatric patients transported to the hospital (75.9% vs. 80.8%; Table 1). It is possible that the increased awareness of caregivers of paediatric patients who called emergency medical technicians (EMTs) resulted in a greater recognition of the seriousness of the symptoms they observed. This was indicated by the observed increase in the number of diagnoses made by EMTs based on the ICD-10 code group Z, which pertained to factors that affected health status and contact with health services. It was also possible that obtaining medical assistance at an appropriate healthcare facility presented certain limitations and difficulties. A Finnish study conducted by Oulasvirta et al. also found that paediatric patients were transported to hospitals less frequently during the pandemic (20).

The current study is based on the data acquired from the nationwide, integrated State Emergency Medical Service Command Support System, which is undoubtedly its major advantage. However, further in-depth research is needed to analyse the impact of the COVID-19 pandemic on the health situation of specific patient groups and the functioning of the healthcare system.

## 5 Conclusion

The COVID-19 pandemic brought about changes in the characteristics of potential health emergencies in children. Based on the conducted analyses, it can be concluded that during the pandemic, there was a decrease in the number of cases related to health emergencies. The age of paediatric patients who received medical assistance decreased. During the pandemic, there was a decrease in the number of patients who were transported to hospitals. Additionally, there were fewer patients who sustained injuries, including head injuries. However, the number of interventions due to fever and related to alcohol consumption by minors increased.

This study highlights the changing health needs of paediatric patients during the COVID-19 pandemic, providing valuable insights for emergency healthcare management.

## Data Availability

The raw data supporting the conclusions of this article will be made available by the authors, without undue reservation.
